# Combined diagnostic value of rheumatoid arthritis-specific citrullinated protein, haemoglobin-to-red cell distribution width ratio, and anti-CCP antibody in rheumatoid arthritis

**DOI:** 10.1080/07853890.2026.2634440

**Published:** 2026-02-23

**Authors:** Xuan Zhang, Junhong Li, Xiaodan Tan, Zhixiao Wei

**Affiliations:** Department of Nuclear Medicine, The First Affiliated Hospital of Guangxi Medical University, Nanning, China

**Keywords:** Rheumatoid arthritis, citrullinated proteins, haemoglobin, red blood cell distribution width, mean platelet volume, rheumatoid arthritis-specific citrullinated protein, Hb/RDW ratio, MPV/PC ratio

## Abstract

**Objective:**

To explore the clinical significance of new haematological indicators, including rheumatoid arthritis-specific citrullinated protein (RA-CP), haemoglobin-to-red cell distribution width ratio (HRR), and mean platelet volume/platelet count (MPV/PC) ratio, in rheumatoid arthritis (RA). This study is the first to evaluate the combined diagnostic utility of RA-CP and HRR in RA.

**Methods:**

Data from 700 subjects, including RA patients, patients with other autoimmune diseases, and healthy controls, were retrospectively analyzed. The relationships between these indicators and the risk of RA were assessed using logistic regression, correlation, and receiver operating characteristic (ROC) curve analyses.

**Results:**

There were significant differences in RA-CP, HRR, and MPV/PC among the three groups (*p* < 0.001, for all). Logistic regression analysis indicated RA-CP and HRR as independent predictors of RA (*p* < 0.001, for both). Moreover, RA-CP and HRR were closely associated with the clinical characteristics. In addition, ROC curve analysis revealed that the combined detection of RA-CP, HRR, and anti CCP antibody achieved the highest area under the ROC curve (AUC_RA - CP + HRR + antiCCP antibody_ = 0.998) for the diagnosis of RA and also improved the diagnostic sensitivity (98.31%).

**Conclusion:**

RA-CP and HRR were identified as independent predictors of RA, including RA-CP as a risk factor for RA and HRR as a protective factor for RA. Combined detection of RA-CP, HRR, and anti-CCP antibody could enhance the accuracy and sensitivity of laboratory diagnosis. Although MPV/PC did not demonstrate significant independent predictive value in this study, this represents the first exploration of its potential clinical role in RA.

## Introduction

Rheumatoid arthritis (RA) is a systemic autoimmune disease marked by pain and swelling in multiple joints and, in some cases, involvement of other organs [1]. It mainly causes chronic and destructive synovial lesions. These lesions are irreversible and disabling, often leading to joint deformity and loss of function [2]. RA is prevalent worldwide [3,4], with reported prevalence ranging from 0.24% to 2% [5–7]. The pathogenesis of RA remains incompletely understood, and no curative treatment is currently available [8,9].

The clinical manifestations of RA are heterogeneous, and there is no single diagnostic method with universal significance. This makes early diagnosis especially difficult [10]. Delayed diagnosis and treatment can severely impair patients’ quality of life [11]. Evidence indicates that ‘early detection, early diagnosis, and early treatment’ are critical for controlling disease progression [12,13].

The combined detection of serum markers is of great significance in the early diagnosis of RA. Anti-cyclic citrullinated peptide (anti-CCP) antibody is a traditional biomarker recommended by international guidelines for RA [14,15]; however, due to individual variability, some patients may not produce this antibody, which may lead to missed diagnosis [16–18].

The Rheumatoid arthritis-specific citrullinated protein (RA-CP) is a product of abnormal immune responses triggered by citrullination, resulting from structural changes in proteins. As a post-translationally modified protein specifically associated with RA, RA-CP plays a role in disease pathogenesis and can be detected in patient serum during the early stages of RA.

RA is often linked to inflammatory anaemia. This is mainly caused by long-term systemic inflammation, which impairs blood cell production and disrupts iron metabolism: RA-related chronic inflammation is the direct cause of anaemia, and anaemia in turn can exacerbate the inflammatory response and disease progression of RA [19,20]. Haemoglobin-to-red cell distribution width ratio (HRR) is a composite parameter calculated by two indicators of haemoglobin (Hb) and red cell distribution width (RDW). It indicates the severity of anaemia and the variability in erythropoiesis, serving as a dual indicator of anaemia and inflammation [19,21]. HRR has been proposed as a novel biomarker for chronic inflammatory diseases, with reported clinical value in the diagnosis [22], condition evaluation [23] and prognosis [24,25] of a variety of diseases.

The MPV/PC ratio is obtained by dividing mean platelet volume (MPV) by platelet count (PC) in routine blood tests. MPV indicates platelet size, while platelet count reflects the total number of platelets. This parameter integrates platelet size heterogeneity and quantitative changes, providing valuable information on platelet function. MPV/PC has demonstrated clinical significance in the diagnosis of platelet related diseases [26,27] and in monitoring the progression of various systemic diseases [28,29].

Therefore, the present study aimed to investigate the clinical significance of RA-CP, HRR, and MPV/PC in RA, and to evaluate the diagnostic efficacy of these markers in combination with the internationally recommended biomarker, anti-CCP antibody.

## Patients and methods

### Patients

**Figure 1.**
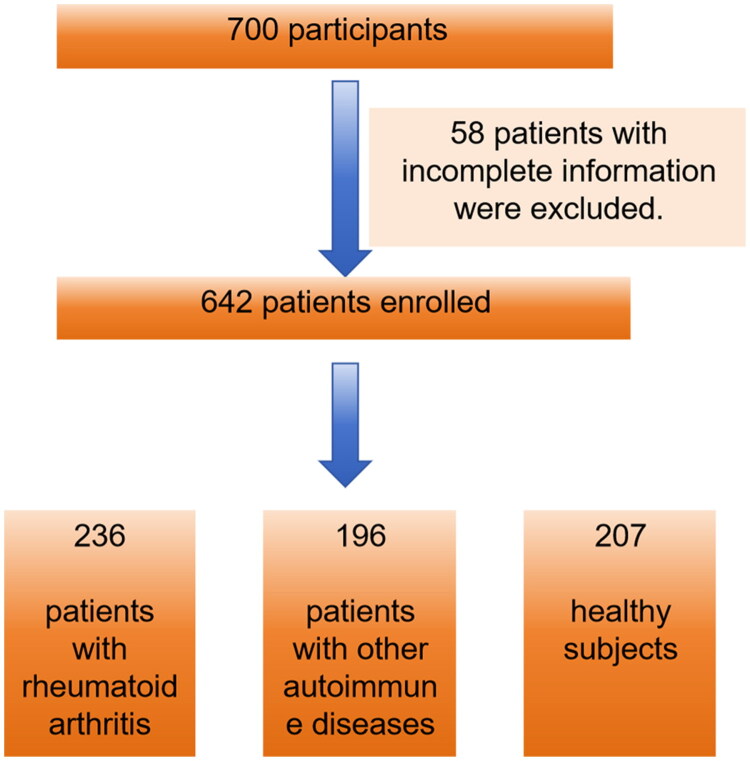
Patient flow chart diagram.

In this retrospective study, a total of 700 participants were included, and 58 subjects with lack of medical records and incomplete information on other parameters were excluded, leading to finally involvement of 642 patients. These subjects were divided into three groups according to the requirements: 236 patients with RA were classified as the study group, the remaining 196 patients with other autoimmune diseases were classified as the disease group, and 207 individuals undergoing routine physical examinations served as the control group (Figure 1). All demographic information and blood test results were obtained from the First Affiliated Hospital of Guangxi Medical University (Nanning, China) between October 2022 and December 2024, and these data were retrospectively analysed in August 2025. Data collected from participants in the study group included age, sex, medical history, and smoking status. The diagnosis of RA was established by clinicians based on a comprehensive evaluation of clinical symptoms, imaging findings, and laboratory test results (European Alliance of Associations for Rheumatology) [30]. Participants were excluded if they met any of the following conditions: (1) pregnancy, (2) lactation, (3) malignancy, or (4) Infection. The majority of patients in the disease group were diagnosed with systemic lupus erythematosus, osteoarthritis, or Sjögren’s syndrome. This study was approved by the Ethics Committee of the First Affiliated Hospital of Guangxi Medical University (Nanning, China; Approval No. 2025-S0441).

### Methods

Fasting venous blood samples were collected from all participants between 6:00 and 9:00 am, with those from the RA group obtained prior to treatment initiation. Whole blood was obtained using K2-EDTA tubes, while serum samples were collected in coagulation-promoting tubes.

Serum RA-CP level was determined using ELISA kits (Guangzhou Lead Biological Technology Co., Ltd., Guangzhou, China) on an Aikon AE65 fully automated enzyme immunoassay analyzer (Zhejiang, China). RACP quality control employs quality control materials provided with the reagent kit, including positive controls, critical controls, and negative controls. For this assay, the absorbance ratio is calculated as the sample OD value (S) divided by the critical control OD value (CO). The gray zone of the assay is defined as 0.95–1.05 (S/CO), and the coefficient of variation (CV) of the critical control OD value is less than 10%.

Routine blood parameters were measured using the Beckman Coulter LH 780 analyzer (Beckman Coulter, Brea, CA, USA). MPV/PC ratio and HRR were calculated from blood cell counts. Erythrocyte sedimentation rate (ESR) was measured using the Minitor-100 automatic analyzer (Electa Lab S.r.l., Forli, Italy), while C-reactive protein (CRP) and Rheumatoid factor (RF) were assessed *via* the 7600-120 Automatic Biochemical analyzer (Hitachi High Technologies, Tokyo, Japan). Anti-CCP antibodies were tested on the IFlash 3000-C chemiluminescent immunoassay analyzer (YHLO Biotech Co., Ltd., Shenzhen, China). All blood samples were processed within 2 h of collection. All procedures were performed in strict accordance with standardized protocols by certified laboratory technicians.

We also assess joint tenderness and swelling in RA using the standardized EULAR criteria for disease activity. The positive rate of haematological indicators in RA patients is calculated as the ratio of RA-CP positive patients to the total number of enrolled RA patients (*n* = 236), and expressed as a percentage (%).

### Statistical analysis

All statistical analyses were performed in SPSS 20.0 (IBM, Armonk, NY, USA). Continuous variables are reported as mean ± standard deviation, with normality checked using the Shapiro–Wilk test. Intergroup comparisons were carried out via one-way ANOVA, with LSD post-hoc tests for pairwise contrasts. We used Spearman correlation and binary logistic regression to analyze the relationships between key indicators and RA, adjusting for relevant risk factors. Use ROC curve to analyze the diagnostic efficacy of RA group and control group, including sensitivity, specificity and cut-off value by MedCalc 18.0 (MedCalc Software Ltd., Ostend, Belgium). A *P*-value < 0.05 was considered statistically significant.

## Results

A total of 642 participants, who were admitted to the First Affiliated Hospital of Guangxi Medical University between October 2022 and December 2024, were enrolled in this retrospective study. Among them, the study group included 236 RA patients, whose age was 52.86 ± 11.98 years old, and women accounted for the majority (76.69%). The age of the disease group (196 people) was 51.57 ± 14.16 years old, and 68.37% were female. The age of the control group (207 people) was 53.16 ± 11.70 years old, and female accounted for 69.57%.

### Demographic and laboratory data: RA, other autoimmune diseases and healthy controls

**Figure 2.**
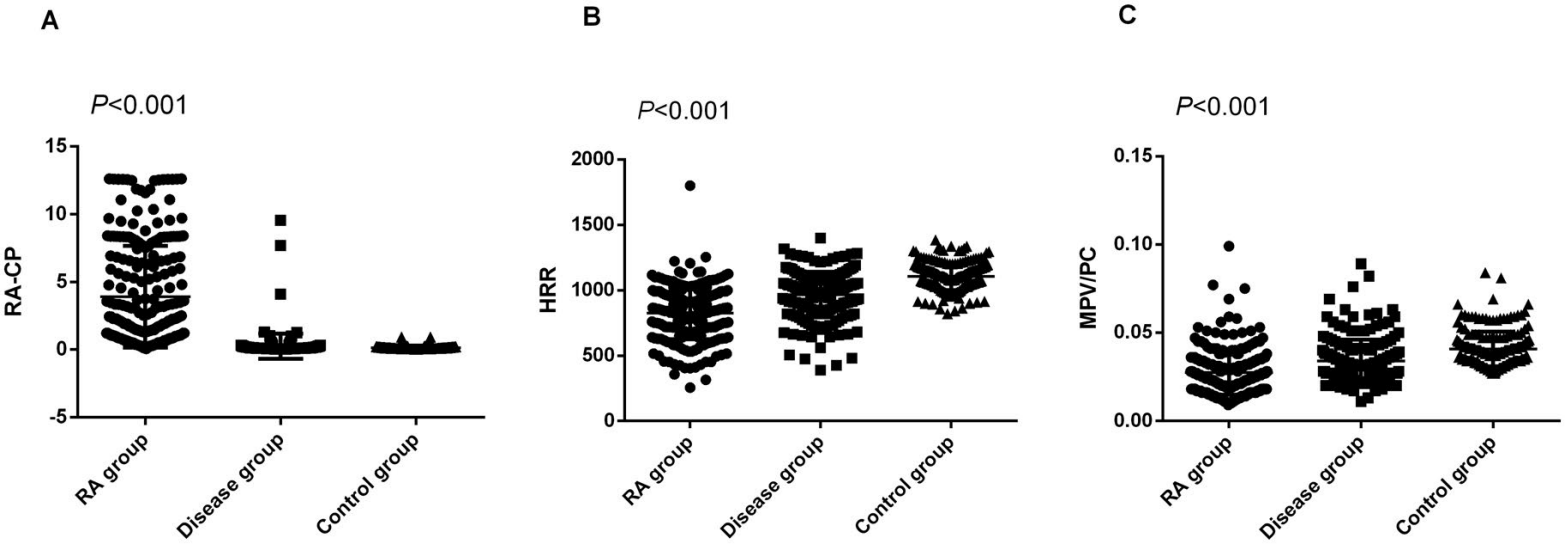
The levels of RA-CP (**A**), HRR (**B**) and MPV/PC (**C**) in patients with rheumatoid arthritis, other autoimmune diseases, and healthy subjects. HRR: Haemoglobin to red cell distribution width ratio; MPV/PC, Mean platelet volume/platelet count ratio; RA-CP, Rheumatoid arthritis-specific citrullinated protein.

As presented in Table 1, significant differences in haematological test indices were identified among the three groups. The markers with statistical significance included RA-CP (*p* < 0.001), anti-CCP antibody (*p <* 0.001), RF (*p <* 0.001), ESR (*p <* 0.001), CRP (*p <* 0.001), HRR (*p <* 0.001), haemoglobin (*p <* 0.001), red cell distribution width (RDW, *p <* 0.001), MPV/PC (*p <* 0.001), albumin (*p <* 0.001), alkaline phosphatase (ALP, *p* = 0.005), prealbumin (*p <* 0.001), cholinesterase (*p <* 0.001), urea (*p* = 0.012), creatinine (*p <* 0.001), uric acid, (*p <* 0.001), immunoglobulin G (IGG, *p <* 0.001), immunoglobulin A (IGA, *p <* 0.001), immunoglobulin M (IGM, *p <* 0.001), platelet (PLT) count (*p <* 0.001) and MPV (*p <* 0.001). Among them, the commonly used test indicators for RA diagnosis, including anti CCP antibody, RF, ESR, and CRP, were the highest in RA group compared with the other two groups (*p <* 0.001, all). Similarly, RA-CP levels in the RA group were also higher than those in the disease and control groups (*p <* 0.001, Figure 2A), while HRR and MPV/PC levels were the smallest among the three groups (*p <* 0.001 (for both), Figure 2B,2C).

**Table 1.** Demographic and laboratory data in patients with rheumatoid arthritis (RA group), other autoimmune diseases (disease group), and healthy subjects (control group).

Parameters	RA group	Disease group	Control group	F	*P*
Number	236	196	207		
Age(year)	52.86 ± 11.98	51.57 ± 14.16	53.16 ± 11.70	0.91	0.403
Sex(femal,%)	181,76.69	134, 68.37	144,69.57	2.23	0.109
RA-CP	3.91 ± 3.76	0.27 ± 0.92^a^	0.15 ± 0.13^b^	185.53	<0.001
Anti-CCP antibody,U/ml	120.86 ± 81.36	1.22 ± 6.42^a^	0.25 ± 0.45^b^	436.93	<0.001
RF,IU/mL	90.33 ± 64.65	9.33 ± 17.00^a^	5.26 ± 3.43^b^	314.38	<0.001
ESR,mm/h	49.83 ± 26.10	20.86 ± 17.39^a^	10.55 ± 4.48^b^	264.32	<0.001
CRP,mg/L	28.58 ± 35.24	6.58 ± 16.60^a^	1.41 ± 1.51^b^	85.64	<0.001
HRR,g/(L·%)	827.95 ± 201.95	968.12 ± 173.76^a^	1106.73 ± 106.13^b^	153.36	<0.001
Hemoglobin,g/L	117.72 ± 17.86	131.25 ± 15.64^a^	145.77 ± 10.91^b^	187.42	<0.001
RDW,	0.147 ± 0.02	0.138 ± 0.02^a^	0.132 ± 0.01^b^	41.78	<0.001
MPV/PC	0.027 ± 0.01	0.034 ± 0.01^a^	0.041 ± 0.01^b^	72.86	<0.001
Albumin,g/L	37.04 ± 4.10	41.12 ± 3.48^a^	43.87 ± 2.22^b^	227.90	<0.001
ALP,U/L	79.68 ± 32.21	79.06 ± 32.51	71.62 ± 16.66^b^	5.33	0.005
Prealbumin,mg/mL	235.45 ± 65.88	284.36 ± 64.00^a^	298.38 ± 52.43^b^	65.11	<0.001
Cholinesterase,U/L	7635.00 ± 1780.73	8733.07 ± 1851.98^a^	8537.77 ± 1640.24^b^	24.56	<0.001
Urea,mmol/L	4.90 ± 1.93	4.96 ± 1.83	5.35 ± 1.14^b^	4.48	0.012
Creatinine,μmol/L	61.78 ± 16.34	69.05 ± 20.24^a^	70.00 ± 14.57^b^	15.44	<0.001
Uric acid,μmol/L	280.80 ± 82.38	332.65 ± 106.26^a^	313.10 ± 56.62^b^	21.31	<0.001
IGG,g/L	18.49 ± 5.90	15.57 ± 4.54^a^	13.02 ± 2.67^b^	77.31	<0.001
IGA,g/L	3.23 ± 1.12	2.46 ± 0.98^a^	2.45 ± 0.83^b^	45.49	<0.001
IGM,g/L	1.76 ± 0.98	1.51 ± 0.71^a^	1.07 ± 0.21^b^	50.60	<0.001
PLT,10^∧^9	347.90 ± 123.29	272.76 ± 71.09^a^	246.18 ± 48.87^b^	78.60	<0.001
MPV,fl	8.26 ± 1.12	8.53 ± 1.16^a^	9.60 ± 0.46^b^	113.61	<0.001

*Note.* Data are expressed as mean ± standard deviation.

^a^Indicates patients with RA vs. patients with other autoimmune diseases: *p <* 0.05 (Tukey’s test).

^b^Indicates patients with RA vs. patients with healthy subjects: *p <* 0.05 (Tukey’s test). The unlabeled data values suggest that no statistically significant differences were observed.

ALP, Alkaline phosphatase; Anti-CCP antibody, Anti-cyclic peptide containing citrulline; CRP, C-reactive protein; ESR, Erythrocyte sedimentation rate; HRR: Haemoglobin to red cell distribution width ratio; IGA, Immunoglobulin A; IGG, Immunoglobulin G; IGM, Immunoglobulin M; MPV, mean platelet volumes; MPV/PC, Mean platelet volume/platelet count ratio; PLT, Platelets; RA-CP, Rheumatoid arthritis-specific citrullinated protein; RDW, Red cell distribution width; RF, Rheumatoid factor.

### The positive rate of each index in the RA group

The positive rates of RA patients are presented in Table 2. Among RA patients positive for both anti-CCP antibody and RF, the detection rates of RA-CP were 98.03% and 97.69%, respectively, higher than ESR (85.22%, 86.11%) and CRP (72.91%, 74.54%). Notably, RA-CP still showed high positive rates (90.91% and 90.00%) in RA patients negative for both anti-CCP antibody and RF, higher than ESR (81.82%, 70.00%) and CRP (60.61%, 30.00%). Our results show that RA-CP can be detected in the serum of most RA patients regardless of anti-CCP antibody or RF status, highlighting its clinical significance in the diagnosis of RA.

**Table 2.** Positive rates of RA-CP, anti-CCP antibody, RF, ESR, and CRP in patients with RA.

Parameters status	N	RA-CP N (%)	Anti-CCP antibody N (%)	RF N (%)	ESR N (%)	CRP N (%)
Total RA cohort	236	229(97.03)				
Anti-CCP antibody	236					
Positive	203	199(98.03)		195(96.06)	173(85.22)	148(72.91)
Negative	33	30(90.91)		21(63.64)	27(81.82)	20(60.61)
RF	236					
Positive	216	211(97.69)	195(90.28)		186(86.11)	161(74.54)
Negative	20	18(90.00)	8(40.00)		14(70.00)	6(30.00)

Abbreviations: CRP, C-reactive protein; ESR, Erythrocyte sedimentation rate; RA-CP, Rheumatoid arthritis-specific citrullinated protein; RF, Rheumatoid factor.

### Binary logistic regression analysis of RA-CP, HRR and MPV/PC in patients with RA and healthy controls

Binary logistic regression analysis was applied to evaluate the relationship between haematological indices and participants. (Table 3). Univariate analysis identified RA-CP, HRR, and MPV/PC ratio as significantly associated with RA (all *p <* 0.001). Multivariate logistic regression analysis confirmed RA-CP and HRR as independent predictors of RA (both *p <* 0.001), with RA-CP serving as a risk factor and HRR acting as a protective factor for RA.

**Table 3.** Binary logistic regression analysis of RA-CP, HRR and MPV/PC in patients with RA and healthy controls.

Parameters	B	SE	Wald χ^2^	*P*	Exp (B)	OR (95% CI)
Univariate logistic regression						
Age	0.01	0.01	0.07	0.79	1.00	0.98-1.01
Sex	0.36	0.22	2.86	0.09	1.44	9.43-2.20
RA-CP	9.10	1.12	66.34	<0.001	8906.56	998.34-79458.61
HRR	−0.01	0.01	105.76	<0.001	0.99	0.99-0.99
MPV/PC	−112.31	12.11	86.05	<0.001	<0.001	0.00-0.00
Multivariate logistic regression						
RA-CP	9.84	1.48	44.54	<0.001	18789.01	1044.15-338100.79
HRR	−0.01	0.01	26.08	<0.001	0.99	0.99-0.99
MPV/PC	−9.42	20.63	0.21	0.65	<0.001	0.00-2.94

Abbreviations: B, Regression coefficient; CI, Confidence interval. HRR: Haemoglobin to red cell distribution width ratio; MPV/PC, Mean platelet volume/platelet count ratio; OR, Odds ratio; RA-CP, Rheumatoid arthritis-specific citrullinated protein; SE, Standard error; Wald χ^2^, Wald Chi-Square Test.

### Correlations of RA-CP, HRR with parameters in RA patients

**Figure 3.**
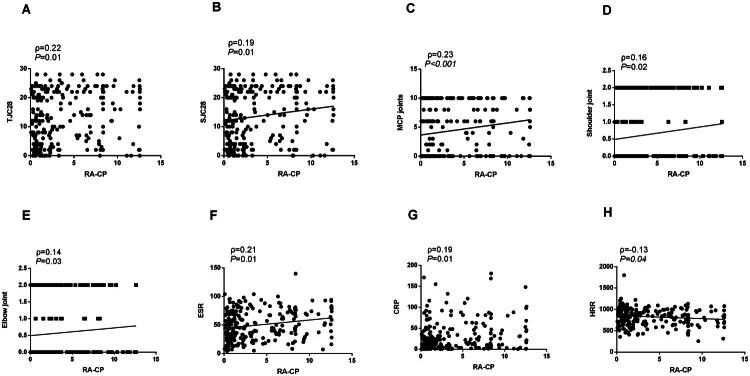
The correlation between RA-CP and clinical characteristics and other parameters in patients with rheumatoid arthritis. RA-CP, Rheumatoid arthritis-specific citrullinated protein.

**Figure 4.**
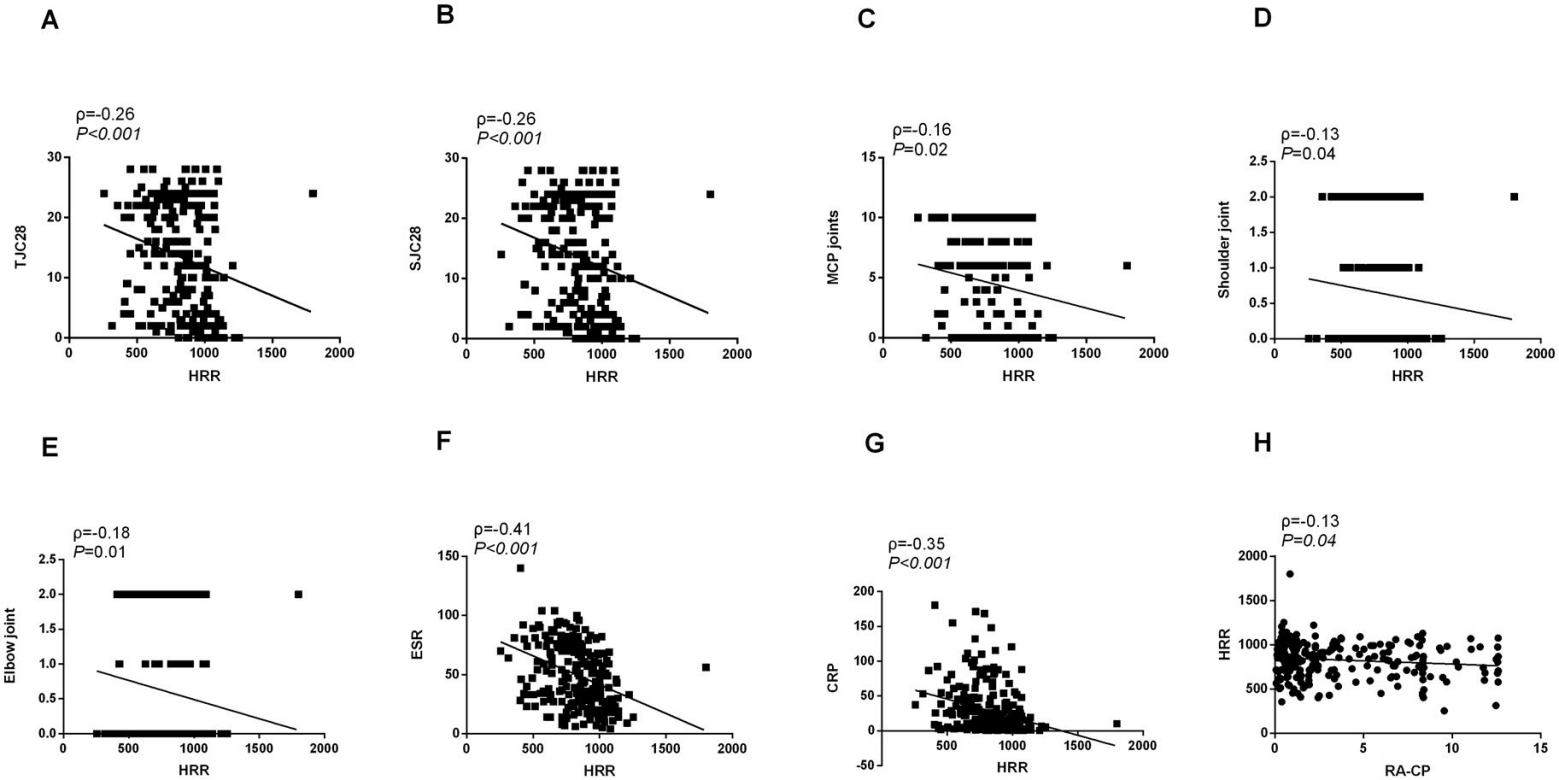
The correlation between HRR and clinical characteristics and other parameters in patients with rheumatoid arthritis. HRR: Haemoglobin to red cell distribution width ratio.

Table 4 presents the correlation analysis among the haematological test indices, clinical characteristics, and RA. Spearman correlation analysis indicated a correlation of RA-CP and HRR with several clinical features: Tender Joint Count 28 (*p* = 0.01, ρ = 0.22, Figure 3A; *p <* 0.001, ρ = –0.26, Figure 4A), Swollen Joint Count 28 (*p* = 0.01, ρ = 0.19, Figure 3B; *p <* 0.001, ρ = –0.26, Figure 4B), metacarpophalangeal Joints(*p <* 0.001, ρ = 0.23, Figure 3C; *p* = 0.02, ρ = –0.16, Figure 4C), shoulder joints (*p* = 0.02, ρ = 0.16, Figure 3D; *p* = 0.04, ρ = –0.13, Figure 4D), and elbow joints (*p* = 0.03, ρ = 0.14, Figure 3E; *p* = 0.01, ρ = –0.18, Figure 4E). Additional correlations included ESR (*p* = 0.01, ρ = 0.21, Figure 3F; *p <* 0.001, ρ = –0.41, Figure 4F), CRP (*p* = 0.01, ρ = 0.19, Figure 3G; *p <* 0.001, ρ = –0.35, Figure 4G), and HRR (*p* = 0.04, ρ = –0.13, Figure 3H and Figure 4H). Furthermore, RA-CP was associated with Heberden’s nodes (*p* = 0.01, ρ = –0.20), anti-CCP antibody (*p <* 0.001, ρ = 0.37), and RF (*p <* 0.001, ρ = 0.25). Moreover, HRR showed correlations with proximal interphalangeal joints (*p <* 0.001, ρ = –0.27), wrist joints (*p <* 0.001, ρ = –0.23), knee joints (*p <* 0.001, ρ = –0.23), and morning stiffness duration (*p <* 0.001, ρ = –0.21).

**Table 4.** The correlation between RA-CP, HRR and clinical characteristics and other parameters in patients with rheumatoid arthritis.

	RA-CP		HRR	
Parameters	ρ	*P*	ρ	*P*
Smoking status	−0.01	0.88	−0.07	0.30
Past medical history status	0.11	0.10	0.03	0.67
Clinical characteristics				
Tender Joint Count 28	0.22	0.01	−0.26	<0.001
Swollen Joint Count 28	0.19	0.01	−0.26	<0.001
Joint swelling and pain				
PIP	0.12	0.06	−0.27	<0.001
Metacarpophalangeal Joints	0.23	<0.001	−0.16	0.02
Wrist joint	0.10	0.12	−0.23	<0.001
Knee joint	0.08	0.24	−0.23	<0.001
Shoulder joint	0.16	0.02	−0.13	0.04
Elbow joint	0.14	0.03	−0.18	0.01
Hip joint	−0.08	0.20	0.04	0.53
Ankle joint	0.09	0.16	−0.15	0.03
Metatarsophalangeal joint	0.08	0.22	−0.10	0.12
Distal Interphalangeal Joints	−0.09	0.15	−0.10	0.14
Joint deformity	0.01	0.83	−0.05	0.44
Morning stiffness time	0.07	0.29	−0.21	<0.001
Heberden’s nodes	−0.20	0.01	0.86	0.86
Age	0.10	0.14	−0.03	0.66
RA-CP			−0.13	0.04
HRR	−0.13	0.04		
Anti-CCP antibody	0.37	<0.001	−0.02	0.74
Rheumatoid factor	0.25	<0.001	0.05	0.44
ESR	0.21	0.01	−0.41	<0.001
C-reactive protein	0.19	0.01	−0.35	<0.001
IGG	−0.01	0.98	−0.07	0.32
IGA	0.11	0.09	−0.02	0.80
IGM	0.18	0.01	−0.10	0.13

Abbreviations: ESR, Erythrocyte sedimentation rate; HRR: Haemoglobin to red cell distribution width ratio; IGA, Immunoglobulin A; IGG, Immunoglobulin G; IGM, Immunoglobulin M; PIP, Proximal Interphalangeal Joints; RA-CP, Rheumatoid arthritis-specific citrullinated protein.

### Receiver operating characteristic curve analysis

**Figure 5.**
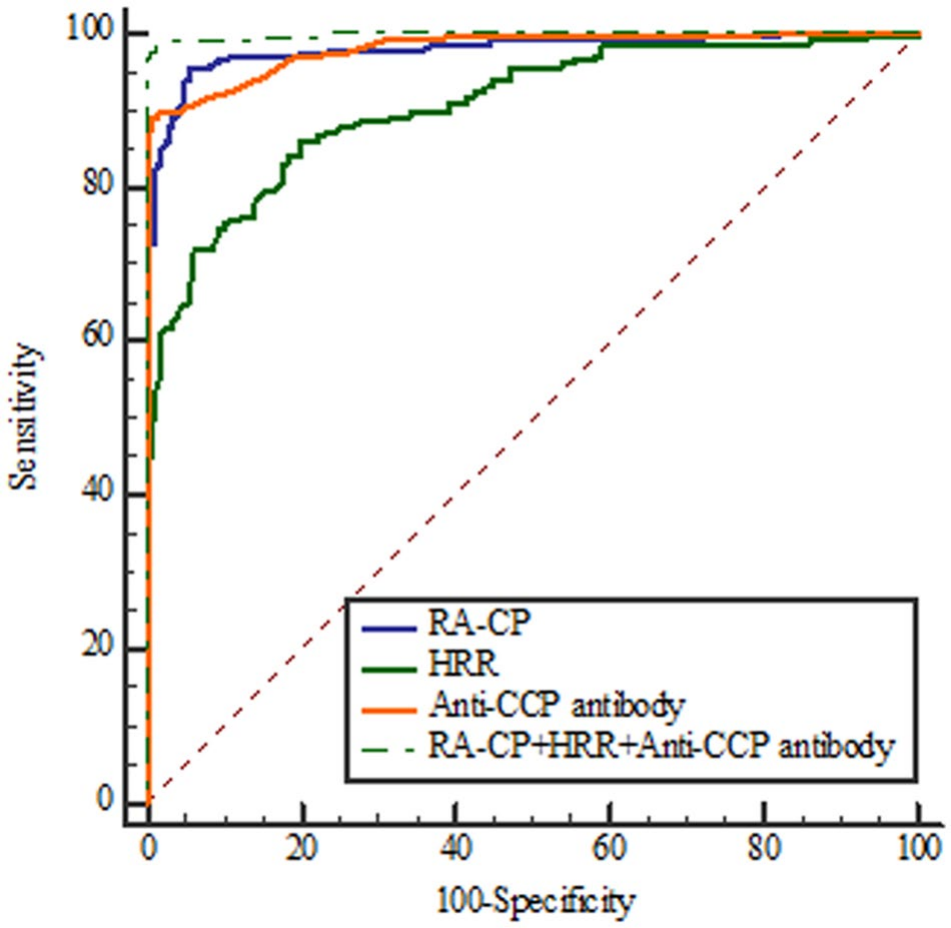
The ROC curve analysis results for RA-CP, HRR and Anti-CCP antibody. Anti-CCP antibody, Anti-cyclic peptide containing citrulline; HRR: Haemoglobin to red cell distribution width ratio; RA-CP, Rheumatoid arthritis-specific citrullinated protein; ROC curve, Receiver Operating Characteristic curve.

The ROC curve analysis revealed that the areas under the ROC curve of RA-CP, HRR, and anti-CCP antibody were 0.980, 0.906, and 0.981, respectively (Table 5), exhibiting high diagnostic efficiency. The sensitivities of RA-CP, HRR, and anti-CCP antibody were 95.34%, 86.02%, and 88.98%, and the specificities were 94.69%, 80.19% and 99.52%, respectively. Among them, RA-CP exhibited the highest sensitivity and anti-CCP antibody achieved the highest specificity. Most importantly, the combination of RA-CP, HRR, and anti CCP antibody yielded the highest diagnostic efficiency for RA (AUC_RA-CP + HRR + Anti-CCP antibody_ = 0.998, Figure 5), thereby improving the diagnostic sensitivity (98.31%).

**Table 5.** Receiver operating characteristic curve analysis.

	AUC	Sensitivity %	Specificity %	Cut-off	PPV%	NPV%	95% Cl
RA-CP	0.980	95.34	94.69	>0.28	95.30	94.70	0.962–0.991
HRR	0.906	86.02	80.19	≤1026.67	83.20	83.40	0.875–0.931
Anti-CCP antibody	0.981	88.98	99.52	>2.10	99.50	88.80	0.963–0.991
RA-CP+HRR+Anti-CCP antibody	0.998	98.31	99.03	>0.42	99.10	98.10	0.988–1.000

Abbreviations: AUC = Area under the curve; HRR: Haemoglobin to red cell distribution width ratio; NPV = Negative predictive value; PPV = Positive predictive value; RA-CP, Rheumatoid arthritis-specific citrullinated protein; ROC, Receiver Operating Characteristic curve.

## Discussion

The early diagnosis of RA is still a major challenge because of the lack of clinically useful biomarkers [31]. Existing laboratory indicators exhibit limited sensitivity and specificity, restricting their utility for the timely detection of RA. This study retrospectively analyzed data from 700 subjects to examine the association between laboratory test results and RA, with a particular emphasis on the combined diagnostic performance of RA-CP, HRR, and anti-CCP antibody.

Participants were categorized into RA, autoimmune disease, and control groups. In the RA group, the mean age was 52.86 ± 11.98 years, and women constituted the majority (76.69%), representing an incidence approximately three times higher than that recorded in men. These findings are consistent with those of previous studies, including those reported by Cush et al. [32]. However, previous reports have also identified smoking as a significant risk factor that may exacerbate RA. The discrepancies between these results and the present study may reflect differences in regional populations, demographic characteristics, and cultural or lifestyle factors.

Comparative analysis revealed that RA-CP, HRR, MPV/PC, and anti-CCP antibody differed significantly across the three groups. Patients with RA exhibited the highest levels of RA-CP and anti-CCP antibody, along with the lowest levels of HRR and MPV/PC. In contrast, healthy controls displayed the lowest RA-CP and anti-CCP antibody levels and the highest HRR and MPV/PC levels. These results indicate that elevated RA-CP and anti-CCP antibody, combined with reduced HRR and MPV/PC, are characteristic features of RA. The reduced HRR found in RA patients align with Kuang et al. [33].’s results. The difference is that the study population enrolled by this scholar was derived from the US. HRR also plays a certain role in monitoring RA treatment follow-up. The study by Yetişir et al. [34] showed that RA patients with good response had significantly higher HRR levels after 4 months of anti-tumour necrosis factor-α treatment compared to before treatment, which can be used as a reference indicator for treatment follow-up. This reduction [34] may reflect the dual role of HRR in indicating both inflammatory status and anaemia, thereby capturing pathological changes associated with immune-mediated inflammation. Given that RA is a chronic autoimmune inflammatory disease [35], diminished HRR serves as an indirect marker of ongoing immune activation and inflammatory activity in affected patients. In conclusion, all these studies have revealed a close association between HRR and RA.

It is obvious that RA-CP serves as the target antigen of anti-CCP antibody. The immune response to RA-CP is involved in the typical inflammatory process of RA and promotes disease progression together with anti-CCP antibody. To date, only a limited number of studies have explored the clinical significance of RA-CP in RA. In our study, elevated RA-CP levels were mainly measured in patients with RA, being consistent with Yang et al.’s findings [36].

In addition, the detection rate of RA-CP exceeded 95% in RA patients who were positive for both anti-CCP antibody and RF. Surprisingly, in RA patients negative for both anti-CCP antibody and RF, the detection rate of RA-CP remained above 90%. These findings suggest that RA-CP can be detected in the serum of most RA patients, even among those negative for anti-CCP antibody and RF, supporting its value as an early diagnostic biomarker in this population. This finding aligns with Chen et al.’s result [37], although the present study demonstrated an even higher positive rate of RA-CP in RA patients.

In our binary logistic regression model, the dependent variable was defined as the diagnosis status of rheumatoid arthritis, and the independent variables included Age, Sex, RA-CP, HRR, MPV/PC; this analysis confirmed RA-CP (risk factor) and HRR (protective factor) as independent RA predictors, in line with Kuang et al. [33]. Multivariate logistic regression analysis revealed that MPV/PC did not independently predict the risk of RA. This may be due to medication-induced alterations in platelet parameters and its weaker, less specific signal compared with RA-CP and HRR. Unlike RA-CP (a disease-specific autoantibody) and HRR (inflammatory-haematological marker), MPV/PC is confounded by non-specific factors, limiting its discriminatory power. As a new marker of acute and chronic diseases, MPV/PC has certain clinical value in various diseases [27,38]. However, this study is the first to explore the diagnostic value of MPV/PC in RA. Although the results indicated that MPV and PLT count have limited utility in the laboratory diagnosis of RA, previous studies have reported that RA patients with ankle joint involvement exhibited significantly lower MPV values [39] and higher PLT counts [40], consistent with the findings of the present study. Moreover, we also found that RA-CP and HRR were statistically correlated with joint pain and swelling, but the correlation coefficients were not large. This may not be helpful for the diagnosis of RA, and a large sample size study is needed to confirm this conclusion. Interestingly, RA-CP was negatively correlated with Heberden’s nodes—a classic manifestation of osteoarthritis (OA) rather than RA. This association may reflect disease differentiation or specific patient subset traits, suggesting that RA-CP has diagnostic value in distinguishing OA from RA and highlighting the need for validation in larger cohorts and longitudinal studies. These findings indicate that RA-CP and HRR are closely and reliably linked to disease activity, supporting their potential value in the clinical assessment of RA.

The ROC curve analysis revealed that the AUC values of RA-CP, HRR, and anti-CCP antibody were 0.980, 0.906, and 0.981, respectively. Moreover, RA-CP exhibited the highest sensitivity, and anti-CCP antibody had the highest specificity. The AUC values were further elevated when the three indicators were combined for the diagnosis of RA. Additionally, the sensitivity of joint detection increased. The findings of this study indicated that the combined detection of RA-CP, HRR, and anti-CCP antibody exhibited superior diagnostic efficiency compared with single-marker detection, being consistent with Yang et al.’s [36] finding. However, there are several notable differences between the two studies. Yang et al. concentrated on the differential diagnostic value of RA-CP in combination with three conventional markers (anti-CCP antibody, RF, and anti-keratin antibodies), whereas the present study evaluated the diagnostic performance of RA-CP in combination with the novel marker HRR and the traditional marker anti-CCP antibody. In addition, the study population in the present study was more diverse. Beyond the RA and disease groups, the control group was included to minimize the confounding influence of general health status, thereby allowing for a more accurate identification of disease-specific characteristics and enhancing the robustness of the findings. The disease group also met stricter inclusion criteria, and most participants were newly diagnosed with RA, reducing the potential impact of treatment-related factors. This may account for the higher diagnostic sensitivity of RA-CP found in this study, which exceeded 95%. In contrast to previous studies, this research further examined the correlation between laboratory indicators and clinical manifestations of RA, including joint involvement, providing additional insights into their potential utility for disease evaluation and management.

Collectively, RA-CP has high sensitivity and specificity as a novel antigenic target of international relevance. HRR is associated with joint lesions in RA and can serve as a reference indicator for disease progression, but it requires validation in large populations and confirmation of its underlying pathological mechanism. Additionally, anti-CCP antibody remains a classic and irreplaceable marker in the diagnosis of RA. The combined detection of RA-CP, HRR, and anti-CCP antibody significantly enhances the accuracy of early laboratory diagnosis and provides valuable support for the clinical management and prognostic evaluation.

Several limitations of this study should be noted. 1) This is a retrospective analysis with bias, and no multiple comparison correction was applied in correlation analyses. Thus, larger-sample, multi-center verification is essential. 2) The RA-CP measurement protocol and combined diagnostic model require external validation in independent cohorts to confirm their reliability and clinical utility. 3) The heterogeneous ‘other diseases’ control group may confound differential diagnostic analyses, which dictates our next research direction: investigating RA-CP’s value for RA differential diagnosis.

## Conclusions

This study further identified RA-CP and HRR as independent predictors of RA, with RA-CP functioning as a risk factor and HRR as a protective factor. Combined detection of RA-CP, HRR, and anti-CCP antibody represents a novel diagnostic strategy, improving both sensitivity and efficiency in early disease detection. Although the MPV/PC ratio did not demonstrate independent predictive value in this study, its potential clinical significance in RA necessitates further investigation with the larger sample size and more stringent control of confounding factors. Further prospective validation is warranted to confirm these findings.

## Data Availability

The datasets used and/or analyzed during the current study are available from the corresponding author on reasonable request.
